# In Situ Formed Amorphous Bismuth Sulfide Cathodes with a Self‐Controlled Conversion Storage Mechanism for High Performance Hybrid Ion Batteries

**DOI:** 10.1002/advs.202304146

**Published:** 2023-11-27

**Authors:** Wei Zhang, Yuanhe Sun, Zhiguo Ren, Yuanxin Zhao, Zeying Yao, Qi Lei, Jingying Si, Zhao Li, Xiaochuan Ren, Xiaolong Li, Aiguo Li, Wen Wen, Daming Zhu

**Affiliations:** ^1^ Shanghai Institute of Applied Physics Chinese Academy of Sciences Shanghai 201800 China; ^2^ Shanghai Synchrotron Radiation Facility Shanghai Advanced Research Institute Chinese Academy of Sciences Shanghai 201204 China; ^3^ University of Chinese Academy of Sciences Beijing 100049 China; ^4^ Industrial Research Institute of Nonwovens and Technical Textiles College of Textiles and Clothing Qingdao University Shandong 266071 China

**Keywords:** amorphous materials, aqueous batteries, bismuth sulfide, conversion reactions, self‐controlled conversion

## Abstract

Conversion‐type electrodes offer a promising multielectron transfer alternative to intercalation hosts with potentially high‐capacity release in batteries. However, the poor cycle stability severely hinders their application, especially in aqueous multivalence‐ion systems, which can fundamentally impute to anisotropic ion diffusion channel collapse in pristine crystals and irreversible bond fracture during repeated conversion. Here, an amorphous bismuth sulfide (a‐BS) formed in situ with unprecedentedly self‐controlled moderate conversion Cu^2+^ storage is proposed to comprehensively regulate the isotropic ion diffusion channels and highly reversible bond evolution. Operando synchrotron X‐ray diffraction and substantive verification tests reveal that the total destruction of the Bi─S bond and unsustainable deep alloying are fully restrained. The amorphous structure with robust ion diffusion channels, unique self‐controlled moderate conversion, and high electrical conductivity discharge products synergistically boosts the capacity (326.7 mAh g^−1^ at 1 A g^−1^), rate performance (194.5 mAh g^−1^ at 10 A g^−1^), and long‐lifespan stability (over 8000 cycles with a decay rate of only 0.02 ‰ per cycle). Moreover, the a‐BS Cu^2+^‖Zn^2+^ hybrid ion battery can well supply a stable energy density of 238.6 Wh kg^−1^ at 9760 W kg^−1^. The intrinsically high‐stability conversion mechanism explored on amorphous electrodes provides a new opportunity for advanced aqueous storage.

## Graphical abstract

An amorphous bismuth sulfide formed in situ demonstrates an unprecedentedly self‐controlled moderate conversion Cu^2+^ storage mechanism for aqueous battery. The amorphous structure with robust ion diffusion channels, unique self‐controlled moderate conversion, and high electrical conductivity discharge products synergistically boosts the capacity, rate performance, and long‐lifespan stability (up to 8000 cycles).

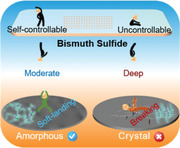

## 1 Introduction

Conversion reaction mechanism for electrodes in rechargeable energy storage devices implies the provision of high theoretical specific capacity and flat voltage plateau compared to ion intercalation and surface redox mechanisms, ensuring an appealing stable energy supply.^[^ ^1^, ^2^, ^3^, ^4^, ^5^, ^6^ ^]^ Driven by the potential high‐performance perspective, intensive efforts have been aimed at developing conversion‐type material electrodes for conventional lithium‐ion batteries and emerging aqueous batteries, including sulfur,^[^ ^7^, ^8^ ^]^ selenium,^[^ ^9^, ^10^ ^]^ tellurium,^[^ ^4^, ^11^ ^]^ and transition metal chalcogenides.^[^ ^12^, ^13^, ^14^, ^15^ ^]^ Nevertheless, the exploration of advanced conversion electrodes has been hindered by its major scientific challenges, especially in aqueous batteries with multivalent carriers; these challenges involve a significant capacity fading with undesirable operating life triggered by structural degradation at the microstructural and chemical bonding levels.^[^ ^16^, ^17^, ^18^ ^]^ At the microstructural level, excessive embedding of large‐scale multivalent ions with destructive Coulomb potential fields expedites the collapse of pristine well‐defined ion diffusion channels and the crushing of anisotropic structures.^[^ ^19^, ^20^, ^21^ ^]^ At the chemical bonding level, deep conversion reactions initiate the complete breakage and reorganization of bonding structures with low reversibility, fundamentally contributing to phase segregation and deterioration of the structural integrity.^[^ ^22^ ^]^ Thus, it is crucial to develop precise strategies to address microstructural disintegration and irreversible evolution of chemical bonds for the development of stable conversion‐type materials with high capacities for aqueous batteries.

For microstructures, tremendous efforts have been dedicated to inhibiting structure degradation by designing functional cladding,^[^ ^23^ ^]^ constructing heterojunctions^[^ ^24^, ^25^ ^]^ and other advanced strategies,^[^ ^26^, ^27^ ^]^ but the collapse of anisotropic ion channels in pristine crystals after long‐term cycling is still inevitable. In this regard, amorphous materials with sustainable isotropic open channels promoting solid‐state ion diffusion offer some additional opportunities.^[^ ^21^, ^28^ ^]^ Amorphous microstructures with both short‐range order and long‐range disorder have been well demonstrated to effectively break the diffusion confinement of topological ordering;^[^ ^29^ ^]^ in addition, these microstructures provide a buffering effect in adapting to lattice strain caused by multivalent ion embedding. However, at the chemical bonding level, bond breakage, side reactions, and irreversible processes caused by repeated conversion reactions (especially overload ion insertion and low‐quality deep alloying upon over‐discharge) fundamentally limit the performance of the conversion‐type cathode, which lacks a sufficient level of stable capacity retention.^[^ ^30^, ^31^ ^]^ For example, destructive deep conversion reactions induce deconstruction of the bismuth sulfide (Bi_2_S_3_) structure either to crystalline nanosheets or amorphous hollow spheres, which only retained 40–53% of capacity after 500 cycles.^[^ ^32^, ^33^ ^]^ Obviously, the design of a synergistic strategy to manipulate both the microstructure and chemical bonding evolution based on novel amorphous materials with controlled moderate conversion mechanisms is essential to meet the future demand for high‐performance conversion‐type electrodes; however, this goal remains elusive.

Herein, we report that Bi_2_S_3_ can be transformed in situ into highly amorphous structures by an electrochemical Cu^2+^ embedding process. The reversible storage capability of Cu^2+^ in aqueous batteries was explored based on transformed amorphous Bi_2_S_3_ (a‐BS), demonstrating an attractive specific capacity of 326.7 mAh g^−1^ at 1 A g^−1^ and outstanding rate performance of 194.5 mAh g^−1^ at 10 A g^−1^. Operando synchrotron X‐ray diffraction (SXRD) and composite ex situ characterizations reveal unprecedented self‐controlled moderate conversion operating in a‐BS. The reserved Bi─S bonds in the reaction and the simultaneous generation of dispersed conductive bismuth monomers in amorphous materials synergistically facilitate fast electron transfer and local chemical electron compensation. Furthermore, the amorphous structure formed in situ and the unprecedented moderate conversion jointly addressed the structural degradation and irreversible bonding evolution faced by the conversion‐type electrode and initiated an excellent cycling stability with a decent capacity decay rate of only 0.02 ‰ per cycle over 8000 cycles at 10 A g^−1^, the highest value in all Bi_2_S_3_‐based batteries. The established a‐BS Cu^2+^‖Zn^2+^ hybrid ion battery delivers durable ion capacity storage performance and a high energy supply of 238.6 Wh kg^−1^ at a power density of 9760 W kg^−1^. The synergistic optimization strategies at the microstructure and bonding levels based on amorphous electrodes with intrinsically high‐stable conversion mechanisms reported here provide insights for the design of conversion‐type electrodes.

## 2 Results and Discussion

**Figure 1:**
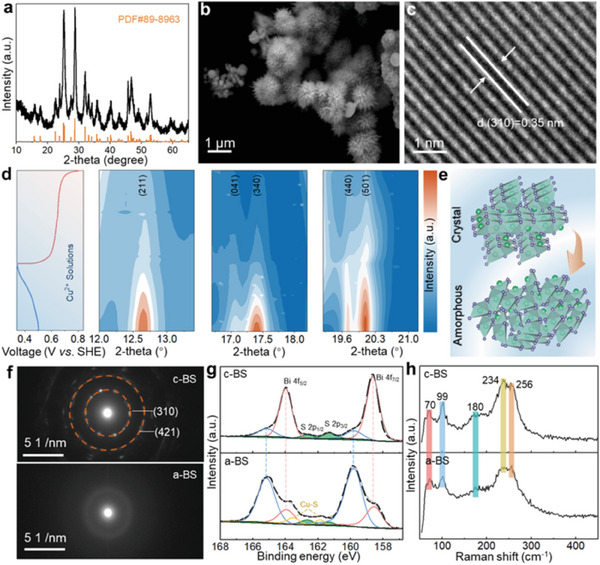
Characterization of c‐BS and a‐BS. a) XRD pattern of c‐BS. b) SEM image of c‐BS at low magnitude. c) HRTEM image of c‐BS. d) The synthesis process of a‐BS by in situ electrochemical activation. e) Schematic diagram of the conversion of c‐BS to a‐BS. f) SAED image of c‐BS and a‐BS. g) XPS spectra of c‐BS and a‐BS. h) Raman spectra of c‐BS and a‐BS.

The crystallized Bi_2_S_3_ (c‐BS) precursors were synthesized by an eco‐friendly one‐step synthesis (Figure S1, Supporting Information). The corresponding X‐ray diffraction (XRD) peaks are well indexed to orthorhombic Bi_2_S_3_ (JCPDS No.89‐8963) in **Figure** **1**a. Scanning electron microscopy (SEM) was used to investigate the morphology of c‐BS, as shown in Figure 1b, which displays a uniform hierarchical nanostructure with a particle size of ≈500 nm (Figure S2, Supporting Information). Energy dispersive X‐ray spectroscopy (EDS) mapping (Figure S3, Supporting Information) was employed to verify the morphology and uniform distribution of Bi and S in c‐BS, and the atomic ratio was close to 2/3 for Bi and S (Figure S4, Supporting Information). The high‐resolution transmission electron microscopy (HRTEM) images of c‐BS in Figure 1c and Figure S5 (Supporting Information) show a lattice distance of ≈0.35 nm, which conforms to the (310) crystal plane. The a‐BS with a large number of defects was further constructed by the electrochemical activation process developed in situ to deform a metastable state containing a large rearrangement of the Bi─S bond during the insertion and extraction process of Cu^2+^ (Figure 1d; Figure S6, Supporting Information). A schematic diagram of the evolution of the crystal structure model is shown in Figure 1e. The selective area electron diffraction (SAED) pattern visually confirms the complete transformation of crystallinity (Figure S7, Supporting Information). The SAED pattern of c‐BS shows two distinct diffraction rings belonging to the (310) and (431) planes with lattice spacings of 3.52 and 1.95 Å, respectively, as displayed in Figure 1f, confirming the high crystallinity of c‐BS and its orthorhombic Pbnm space group. In contrast, the SAED image of a‐BS does not contain any visible diffraction spot features, demonstrating an amorphous configuration. X‐ray photoelectron spectroscopy (XPS) was performed to determine the electronic structure and chemical components of c‐BS and a‐BS. The XPS survey scan image in Figure S8 (Supporting Information) confirms that c‐BS is mainly composed of S and Bi elements, which is consistent with the result of EDS mapping. The high‐resolution XPS spectrum of Bi 4f is shown in Figure 1g. Two strong peaks located at 158.7 and 164 eV can be attributed to Bi 4f_7/2_ and Bi 4f_5/2_, respectively, and split into two shoulders at 159.7 and 165 eV.^[^ ^34^ ^]^ Moreover, the two peaks have a typical 5.3 eV energy difference for the spin‐orbit splitting of the Bi 4f core level, illustrating that the valence state of Bi is +3.^[^ ^35^ ^]^ The weaker peaks at 161.3 and 162.6 eV correspond to S 2p_3/2_ and S 2p_1/2_ as‐signed. Noticeably, c‐BS and a‐BS exhibit the same identical Bi 4f and S 2p core‐level peaks, and only the shoulder peak area ratio is varied, which is ascribed to the disorder rearrangement of the Bi─S bonds in the amorphous structure.^[^ ^36^ ^]^ Trace amounts of Cu─S bonds reveal the induction of amorphous structure formation by Cu^2+^. The bonding structure of c‐BS and a‐BS was further confirmed by Raman analysis, as shown in Figure 1h. The c‐BS was characterized by five distinct vibration peaks at 70, 99, 180, 234, and 256 cm^−1^,^[^ ^37^ ^]^ and a‐BS is essentially the same as c‐BS with the peak positions exhibiting good consistency. Thus, the unique a‐BS was successfully obtained based on an in situ electrochemical activation process that was certified by multiple characterization methods.

**Figure 2:**
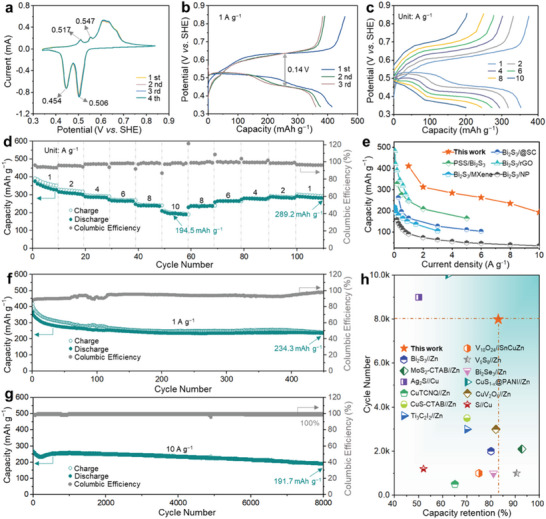
a) CV curves of the a‐BS cathode at a scan rate of 0.4 mV s^−1^. b) Galvanostatic charge‒discharge voltage profiles at 1 A g^−1^ (the first four cycles). c) Galvanostatic charge‒discharge profiles at various current densities: 1–10 A g^−1^. d) Rate performance of the a‐BS‖Cu battery at the indicated current density. e) Comparison of the specific capacity at various current densities between this work and previously reported Bi_2_S_3_ electrodes. f) Cycling performance of the a‐BS cathode at 1 A g^−1^. g) Cycling performance of the a‐BS cathode at 10 A g^−1^. h) Performance comparison of long‐term cycling and capacity retention between this work and previously reported aqueous batteries.

The unique aqueous Cu^2+^‐driven amorphous transformation of Bi_2_S_3_ further prompted the investigation of its performance for reversible storage of Cu^2+^. **Figure** **2**a shows the cyclic voltammetry (CV) curves of a‐BS with a scan rate of 0.4 mV s^−1^ over the voltage range of 0.34–0.84 V versus standard hydrogen electrode (SHE). The first four cycles of the CV curves almost overlap, indicating that the electrochemical reaction in a‐BS is highly reversible. Two pairs of redox peaks positioned at 0.454/0.506 and 0.517/0.547 V can be observed, corresponding to the reversible electrochemical accommodation of Cu^2+^ into a‐BS. The galvanostatic charge/discharge (GCD) profiles (Figure 2b) demonstrate that a‐BS delivers an initial discharge capacity of ≈414.7 mAh g^−1^ at a current density of 1 A g^−1^. Moreover, the GCD curves display two quasi‐discharge plateaus at 0.51 and 0.45 V, which is consistent with the location of reduction peak in the CV curves. In addition, the polarization voltage is ≈0.14 V, much lower than that of other Bi_2_S_3_‐metal ion systems (Li,^[^ ^38^ ^]^ Na,^[^ ^39^ ^]^ K,^[^ ^40^ ^]^ Zn^[^ ^41^ ^]^//Bi_2_S_3_, at a current density of 1 A g−1) (Figure S9, Supporting Information); these results suggest that amorphous Bi2S3 contributes to high ion solid‐phase diffusion kinetics and good interfacial ion/electron transport capability. The GCD curves and corresponding rate performance are shown in Figure 2c,d. The GCD curves at different current densities depict excellent recoverability in Figure 2c. The specific capacities of the a‐BS cathodes at current densities of 1, 2, 4, 6, 8, and 10 A g^−1^ are 326.7, 308.9, 285.5, 265.1, 237.9, and 194.5 mAh g^−1^, respectively. When the current density returned to 1 A g^−1^, the capacity gradually returned to 289.2 mAh g^−1^, demonstrating high reversibility and fast charge storage kinetics. Notably, the rate performance of a‐BS surpasses that of other well‐designed Bi_2_S_3_ electrodes in typical ion battery systems,^[^ ^40^, ^41^, ^42^, ^43^, ^44^ ^]^ as shown in Figure 2e, which can be attributed to the high ionic conductivity of the aqueous electrolyte and the remarkable Cu^2+^ accommodation capability of a‐BS. The long‐term cyclic stability was evaluated at a current density of 1 A g^−1^, as shown in Figure 2f. The a‐BS can deliver a high capacity of 234.3 mAh g^−1^ after 450 cycles. Even at an ultrahigh current density of 10 A g^−1^ (Figure 2g), a‐BS maintains a discharge capacity of 191.7 mAh g^−1^ after 8000 cycles with a Coulombic efficiency (CE) of almost 100%, which far exceeds that of all reported Bi_2_S_3_‐based secondary ion batteries (Table S1, Supporting Information). When conditions are relaxed to mild aqueous batteries with reliable safety, the a‐BS electrode established here also maintains significant superiority in long‐term cycle lifespan performance and capacity retention (Figure 2h).^[^ ^44^, ^45^, ^46^, ^47^, ^48^, ^49^, ^50^, ^51^, ^52^, ^53^, ^54^, ^55^ ^]^ More efforts to evaluate the electrochemical properties of a‐BS electrodes with limit electrolyte quantity (from 200 to 10 µL per cell) or high mass loadings were dedicated, which all demonstrate decent performance (Figures S10 and S11, Supporting Information).

**Figure 3:**
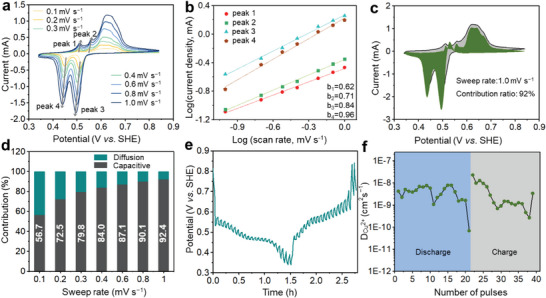
a) CV curves at various sweep rates from 0.1 to 1 mV s^−1^. b) Log (peak current) versus log (sweep rate) plots of the a‐BS‖Cu battery at Peaks 1, 2, 3, and 4. c) CV profile at 1.0 mV s^−1^ indicating the capacitive contribution (green region) to the total current. d) The contribution ratios of the ion diffusion and surface capacitive contribution. e) GITT curves of the a‐BS cathode. f) Cu^2+^ diffusion coefficients (*D*_ions_) of the a‐BS electrode.

The kinetics properties of the a‐BS cathode and Cu^2+^ storage mechanism were subsequently investigated by consecutive CV measurements at diverse scan rates from 0.1 to 1 mV s^−1^ (**Figure** **3**a). The CV curves of Bi_2_S_3_ display a high overlapping shape with different sweep rates, indicating fast kinetics and a small polarization voltage. The peak current (i) and sweep rate (*v*) are subject to the following relationship:

(1) $$i\;=\;av^{b}$$

In this equation, *a* and *b* are constants, in which the value of *b* is correlated with surface‐controlled processes (capacitive behavior) and diffusion‐limited processes (battery behavior). *b* can be derived by fitting the log (i) versus log (*v*) curve with a value between 0.5 and 1. A *b*‐value of 1 indicates capacitive behavior, whereas a value of 0.5 suggests battery behavior. In this work, the fitted *b*‐values are 0.62, 0.71, 0.84, and 0.96 for peaks 1, 2, 3, and 4, respectively (Figure 3b), indicating the coexistence of a synergistic charge storage process. To quantify the capacitance contribution, the following equations:

(2) $$i\left(v\right)\;=k_{1}\;v+k_{2}v^{1/2}$$

or

(3) $$\;i/v^{1/2}=k_{1}\;v^{1/2}+k_{2}$$

are utilized to evaluate the contributions of capacitance (*k*_1_ *v*) and diffusion (*k*_2_ *v* ^1/2^) at a particular scan rate. As shown in Figure 3c, the shaded area of the CV curve fitted at a sweep rate of 1 mV s^−1^ indicates that the capacitance contribution is ≈92.4%. The capacitive contribution gradually rises from 56.7% to 92.4% along with the increase in sweep rate from 0.1 to 1 mV s^−1^, indicating that the capacitive behavior dominates in Figure 3d. Reaction kinetics were probed by the galvanostatic intermittent titration technique (GITT) (Figure 3e). A slight fluctuation in the ion diffusion coefficient (*D*_ion_^2+^) can be observed ranging from 6.76  × 10^−11^ to 7.84 × 10^−9^ and 2.67  × 10^−10^ to 2.28  × 10^−8^ cm^2^ s^−1^ over the entire Cu^2+^ insertion/extraction process, respectively. The high ion diffusion coefficient suggests that a‐BS exhibits a fast ion diffusion capability. This superb kinetics performed in the a‐BS cathode is further confirmed by the low resistances of both charge transfer and Warburg diffusion, as shown in Figure S12 (Supporting Information).

**Figure 4:**
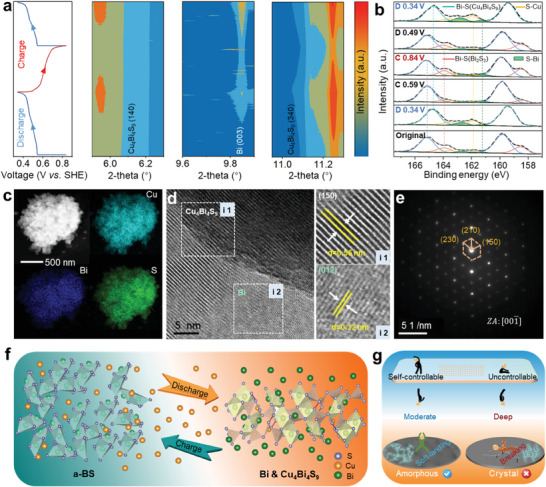
a) Galvanostatic discharge‐charge curve and corresponding 2D contour map of SXRD. b) Ex situ XPS spectrum of Bi 4f and S 2p of a‐BS cathodes at the various discharging–charging states. c) HAADF image and corresponding EDS mappings of a‐BS cathodes in the fully discharged state. d) HRTEM image of a‐BS cathodes in the fully discharged state, square i1 for Cu_4_Bi_4_S_9_, square i2 for Bi. e) SAED image of a‐BS cathodes in the fully discharged state. f) Schematic illustration of the Cu^2+^ storage mechanism of a‐BS. g) Conceptual diagram of the soft landing of self‐controlled moderate conversion versus electrode breaking of uncontrolled deep conversion during discharge

To explore the Cu^2+^ storage mechanism of a‐BS, the structural changes of a‐BS were investigated by operando SXRD (*λ* = 0.6887 Å) during the discharge–charge process. The results are shown in **Figure** **4**a and Figure S13 (Supporting Information), including the 2D contour map of the SXRD patterns and the corresponding discharge–charge curve. During the discharge process, three sets of characterized peaks located at 5.9°, 11.2°, and 9.9° emerged, which are indexed to the (140) and (340) crystal planes of Cu_4_Bi_4_S_9_ and the (003) crystal planes of Bi, respectively. The charge process is the reverse of the discharge process. It can be clearly observed from the 2D contour map of the SXRD patterns that the discharge products Cu_4_Bi_4_S_9_ and Bi disappeared and then returned to the a‐BS phase, namely, a highly reversible moderate conversion reaction occurred between a‐BS and Cu_4_Bi_4_S_9_ as well as Bi. Ex situ XPS analyses of a‐BS electrodes at different states were employed to further elucidate the transformation process and investigate the valence state change of a‐BS electrodes, as shown in Figure 4b. At the two consecutively fully discharged states (D 0 V), there are two obvious peaks corresponding to Bi 4f_5/2_ and Bi 4f_7/2_ at 164.7 and 159.5 eV in a‐BS and remain highly consistent, which are attributed to Cu_4_Bi_4_S_9_.^[^ ^56^ ^]^ Notably, the Bi 4f valence band is noticeably redshifted compared with the original states, which is caused by the blending of the Bi‐metal phase with Cu_4_Bi_4_S_9_ upon the discharge process. After being fully charged to 0.5 V, the Bi 4f double peaks return to high binding energy and represent the a‐BS phase, confirming that the conversion between Cu_4_Bi_4_S_9_ and Bi to a‐BS upon the charge process is highly reversible. Interestingly, total destruction of the Bi─S bond during the entire discharge–charge process was not observed; instead, a trend opposite to the change in intensity of the Cu─S bond formed occurred, which demonstrates a moderate conversion reaction with reserved Bi─S bond. Different from the traditional deep conversion reaction in alkali metal batteries and aqueous batteries, a‐BS undergoes a self‐controlled moderate conversion reaction without an alloy reaction between Bi and Cu upon the discharge process, ensuring high electrical conductivity and cathode activity (a Bi‐loaded CuS composite cathode with a deep conversion as a control group as shown in Figure S14, Supporting Information). In addition, the Cu 2p XPS spectra of a‐BS electrodes at various discharge–charge states are displayed in Figure S15 (Supporting Information). The intensity of Cu 2p regularly increases and decreases during the discharge–charge process with the embedding and release of Cu^2+^, in agreement with the evolution of the Cu─S bond in Figure 4b. Additionally, the binding energy of Cu 2p without shifting indicates that the valence state of Cu ions remains +2 upon the overall discharge–charge process.

HRTEM of the a‐BS electrode in the fully discharged state was carried out to better corroborate the above conversion process. The EDS pattern reveals that Cu, S, and Bi are uniformly distributed on the surface during the discharge process (Figure 4c), suggesting the effective storage of Cu^2+^. Moreover, the HRTEM images reveal the presence of two phases of orthorhombic Cu_4_Bi_4_S_9_ with a lattice spacing of 0.56 nm and rhombohedral Bi with a lattice spacing of 0.32 nm. The SAED pattern of the discharge products shows a defined diffraction spot array with the [00‐1] zone axis, indicating the generated Cu_4_Bi_4_S_9_ with high crystallinity (Figure 4e). After 50 cycles, the HRTEM images display the same discharge species, suggesting the robust self‐controlled conversion process (Figure S16, Supporting Information). Notably, the HRTEM and SAED associated with the fully charged products were observed without any crystalline features, as shown in Figures S17 and S18 (Supporting Information). Based on the above analysis, we proposed that the electrochemical reactions of the a‐BS electrode with Cu^2+^ storage can be identified as a reversible self‐controlled moderate conversion reaction and formulated as Bi_2_S_3_ + Cu^2+^ ⇌ Bi + Cu_4_Bi_4_S_9_ (Figure 4f). Note that the moderate conversion reaction process revealed on amorphous electrodes essentially differs from conventional crystalline cathode intercalation and deep conversion reactions, as shown in Figure 4g. During this self‐controlled moderate conversion process, the total destruction of the Bi─S bond and unsustainable low‐quality deep alloying are fully restrained, while bismuth monomers with high conductivity are generated to synergistically facilitate rapid electron transfer and highly reversible bond evolution.^[^ ^57^, ^58^ ^]^

Encouraged by the highly reversible reaction of a‐BS with Cu^2+^ and the lower electrode potential of the zinc electrode, a high energy density hybrid ion battery was subtly constructed. We employ an anion‐exchange membrane (AEM) to separate the cell into the following compartments: one for the aqueous a‐BS cathode and one for the Zn metal anode. The working mechanism of the a‐BS Cu^2+^‖Zn^2+^ hybrid ion battery is illustrated in Figure S19 (Supporting Information). During the discharge process, Zn^2+^ is stripped from the Zn metal anode, and Cu^2+^ is inserted into a‐BS to form Cu_4_Bi_4_S_9_. The reverse reaction then occurs during the subsequent charging process. To balance the charge neutrality, SO_4_^2−^ anions common in both compartments act as the commuting shuttles between the CuSO_4_ and ZnSO_4_ electrolytes. The reactions during cathode and anode discharge are suggested by the following equation:

(4) $$\mathrm{Cathode}:3Bi_{2}S_{3}+4Cu^{2+}⇌2\mathrm{Bi}+Cu_{4}{\mathrm{Bi}}_{4}S_{9}$$

(5) $$\mathrm{Anode}:\;{\mathrm{Zn}}^{\left(2+\right)}+2e⇌\mathrm{Zn}$$

**Figure 5:**
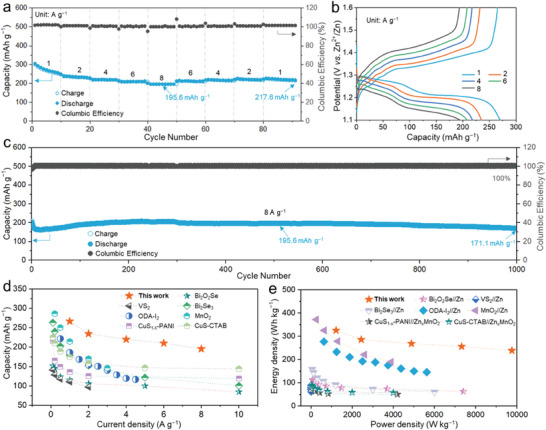
a) Galvanostatic discharging‐charging curves of the a‐BS Cu^2+^‖Zn^2+^ hybrid ion battery at various current densities. b) Galvanostatic charge‐discharge voltage profiles at various current densities: 1–8 A g^−1^. c) Cycling performance of the a‐BS cathode at 8 A g^−1^. d,e) Comparison of the specific capacity and Ragone plot at various current densities between this work and previously reported representative aqueous zinc ion batteries.

Benefiting from the unique amorphous structure and self‐controlled moderate conversion, the assembled hybrid ion battery exhibited distinguished ionic transportation kinetics, resulting in decent rate performance. **Figure** **5**a shows that the reversible capacities of the a‐BS cathode were 266.7, 234.5, 220.1, 210.1, and 195.6 mA h g^−1^ at 1, 2, 4, 6, and 8 A g^−1^, respectively, and the Coulombic efficiency was almost 100%. When the current returns to 1 A g^−1^, the a‐BS cathode could quickly recover a reversible capacity of 227.6 mA h g^−1^ and remain stable. Galvanostatic charge–discharge voltage profiles at various current densities shown in Figure 5b. Profit from the low redox potential of Zn (−0.76 V vs SHE), the aqueous a‐BS Cu^2+^‖Zn^2+^ hybrid ion battery delivers a stable discharging voltage of ≈1.22 V at 1 A g^−1^, while the polarization voltage is maintained at ≈140 mV. Because of the enhanced output voltage, the a‐BS Cu^2+^‖Zn^2+^ hybrid ion battery can achieve a high energy density of 238.6 Wh kg^−1^ at a power density of 9760 W kg^−1^. In addition, the a‐BS Cu^2+^‖Zn^2+^ hybrid ion battery can be easily cycled for more than 1000 cycles at a high current density of 8 A g^−1^ and maintains a specific capacity of 171.1 mAh g^−1^, as shown in Figure 5c. Obviously, the excellent electrochemical performance of the aqueous a‐BS Cu^2+^‖Zn^2+^ hybrid ion battery outperforms the typical cathodes in aqueous zinc ion batteries,^[^ ^46^, ^53^, ^55^, ^59^, ^60^, ^61^, ^62^ ^]^ as shown in Figure 5d. It delivers desired energy densities at admire power densities (Figure 5e).^[^ ^46^, ^53^, ^55^, ^59^, ^60^, ^61^, ^62^ ^]^ The decent performance of the a‐BS Cu^2+^‖Zn^2+^ hybrid ion battery demonstrates its preponderance in amorphous structures along with self‐controlled moderate conversion.

## 3 Conclusion

In this study, we obtained a‐BS with sustainable isotropic open channels by elaborate in situ amorphization of c‐BS. The constructed a‐BS cathode exhibits an unprecedented self‐controlled moderate conversion Cu^2+^ storage capacity, which effectively surmounts the collapse of anisotropic ion diffusion channels in the pristine crystal and irreversible bond fracture during repeated conversion. Operando SXRD and substantial ex situ characterization reveal that the reserved Bi‐S bond during the electrochemical reaction and the dispersed conducting bismuth monomer produced in the cathode material synergistically promote electron transfer and local charge compensation. Consequently, a‐BS delivers a remarkable reversible capacity of 191.7 mA g^−1^ after 8000 cycles at 10 A g^−1^ and a decay rate of only 0.02‰ per cycle. Moreover, the a‐BS Cu^2+^‖Zn^2+^ hybrid ion battery can supply a stable energy density of 238.6 Wh kg^−1^ at 9760 W kg^−1^. This work presents synergistic optimization strategies at the microstructure and bonding levels based on amorphous electrodes with intrinsically high‐stability conversion mechanisms and provides insights for the design of conversion‐type electrodes.

## Conflict of Interest

The authors declare no conflict of interest.

## Supporting information

Supporting Information

## Data Availability

The data that support the findings of this study are available from the corresponding author upon reasonable request.
