# Supplementary material for: In Situ Formed Amorphous Bismuth Sulfide Cathodes with a Self‐Controlled Conversion Storage Mechanism for High Performance Hybrid Ion Batteries
Source: Adv Sci (Weinh). 2023 Nov 27;11(2):2304146. doi: 10.1002/advs.202304146 (PMC10787086; doi:10.1002/advs.202304146)
Supplement: Supplementary file 1 — Supporting Information [file ADVS-11-2304146-s001.pdf]

ADVANCED SCIENCE
Open Access

Supporting Information

for *Adv. Sci.*, DOI 10.1002/advs.202304146

In Situ Formed Amorphous Bismuth Sulfide Cathodes with a Self-Controlled Conversion Storage Mechanism for High Performance Hybrid Ion Batteries

Wei Zhang, Yuanhe Sun, Zhiguo Ren, Yuanxin Zhao, Zeying Yao, Qi Lei, Jingying Si, Zhao Li, Xiaochuan Ren, Xiaolong Li, Aiguo Li, Wen Wen* and Daming Zhu**

Supporting Information

In Situ Formed Amorphous Bismuth Sulfide Cathodes with a Self-Controlled Conversion Storage Mechanism for High Performance Hybrid Ion Batteries

Wei Zhang#, Yuanhe Sun#, Zhiguo Ren#, Yuanxin Zhao, Zeying Yao, Qi Lei*, Jingying Si, Zhao Li, Xiaochuan Ren, Xiaolong Li, Aiguo Li, Wen Wen*, Daming Zhu*

#These authors contributed equally to this work.

*Corresponding authors

Experimental Section

Materials Synthesis

Bi_2S_3 nanosheets were prepared by a hydrothermal method. The necessary chemicals were $Bi(NO_3)_3\cdot 5H_2O$ (99.0%), HCl, and thioacetamide (TAA), which were purchased from Sinopharm Chemical Reagent Co., Ltd. First, 1.87 g $Bi(NO_3)_3\cdot 5H_2O$, 0.96 ml HCl, and 1.31 g TAA were added sequentially in 250 ml of deionized water (DI) and then dissolved uniformly with a magnetic stirrer for 1 h. Subsequently, the solution was transferred to the oven and reacted for 72 h at 60 °C. After that, black viscous deposition was obtained and washed five times in turn with alcohol and DI water. The acquired Bi_2S_3 nanosheet was dried at room temperature and then ground to powder in an agate mortar for approximately 10 minutes.

Material Characterization

The synthesized Bi_2S_3 was characterized by a Bruker D8 Advance X-ray diffractometer using Cu-Kα radiation (λ = 1.54178 Å). Raman spectral tests were performed on a spectrometer (RENISHAW inVia Basis; 532 nm). Transmission electron microscopy (TEM, JEM-2100) was performed to obtain TEM and HRTEM images of the pristine Bi_2S_3 electrode. Ex situ X-ray photoelectron spectroscopy (XPS) spectra were obtained on a Kratos Analytical Axis UltraDLD instrument (Calibrated to reference C 1s peak (284.8 eV)). SXRD experiments were conducted on beamline BL02U2 (X-ray wavelength of 0.6887 Å after adoption of the NIST LaB_6 standard (660b)) at the Shanghai Synchrotron Radiation Facility (SSRF), and the size of the beam was confined to 0.3×0.3 mm^2. The SXRD signal is obtained through the Pilatus 2 M detector and converted into a two-dimensional SXRD signal using Fit2D software integration. Moreover, the custom-built CR2032-coin cell was designed with two 4 mm diameter transparent visual windows in the centre of each positive and negative shell, sealing them with polyimide tape.

Electrochemical Evaluation

To prepare the Bi_2S_3 electrode, Bi_2S_3 powder, acetylene black, and polyvinylidene fluoride (PVDF) (weight ratio of 7:2:1) were mixed in 1-methyl-2-pyrrolidinone solvent with magnet stirring to form a homogeneous slurry for 12 h and then coated

on carbon paper and dried in a vacuum drying oven at 60° for 12 h to obtain a load of approximately 1.7 mg cm^{-2}. An electrochemical activation process was designed for the transformation of c-BS to a-BS state (the c-BS used as working electrode, copper foil as the counter/reference electrode, and 1M $CuSO_4$ aqueous solution as electrolyte). The amorphous activation was conducted at a current density of 200 mA g^{-1} (calculation based on the mass of c-BS), and the fully amorphization of BS can be obtained after one activation cycle. A CR2025 coin battery with a Bi_2S_3 electrode as the cathode, a high-purity Cu foil as the anode, a Whatman glass fibre membrane as the separator and aqueous 1 M $CuSO_4$ as the electrolyte (200 μL) was assembled. Galvanostatic charge and discharge (GCD) measurements were tested on a battery testing system (Neware) with a voltage range of 0-0.5 V vs. Cu/Cu^{2+} and 1.1-1.6 V vs. Zn/Zn^{2+}. Galvanostatic intermittent titration technique (GITT) measurements were tested on a LAND CT2001A battery test system. Cyclic voltammetry measurements were performed on an electrochemical workstation (CHI 760E) at various scan rates. Electrochemical impedance spectroscopy (EIS) was carried out on an electrochemical workstation (AUTOLAB) at frequencies of 100 kHz ~ 0.01 Hz and an AC voltage of 5 mV. The a-BS $Cu^{2+}\|Zn^{2+}$ hybrid ion battery with 1 M $CuSO_4$ electrolyte on the a-BS side and 1 M $ZnSO_4$ electrolyte on the zinc foil side was separated by an anion exchange membrane (AEM) (fumasepFAB-PK-130).

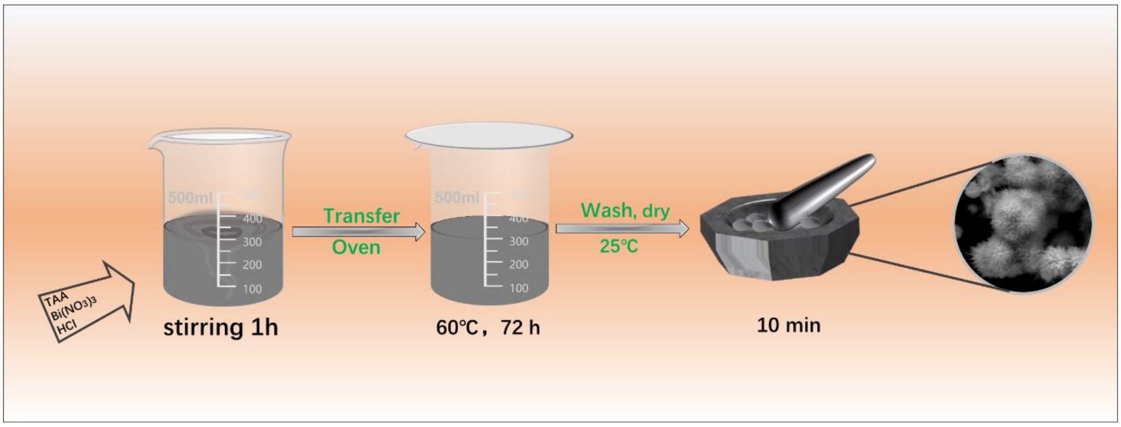

Figure S1. Schematic diagram of the detailed synthetic process of c-BS.

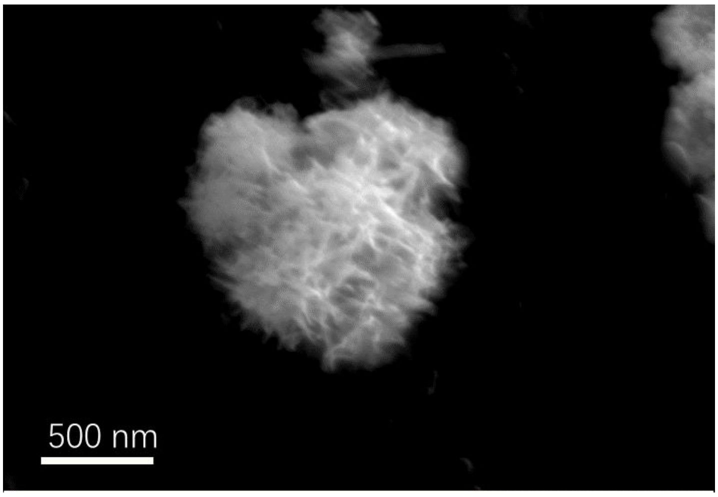

Figure S2. SEM images of c-BS.

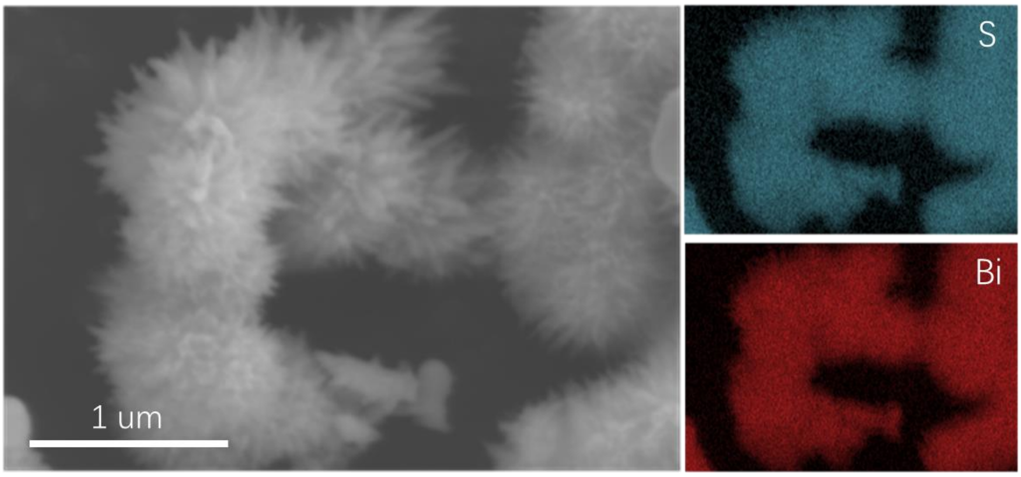

Figure S3. SEM image and corresponding elemental mapping of c-BS.

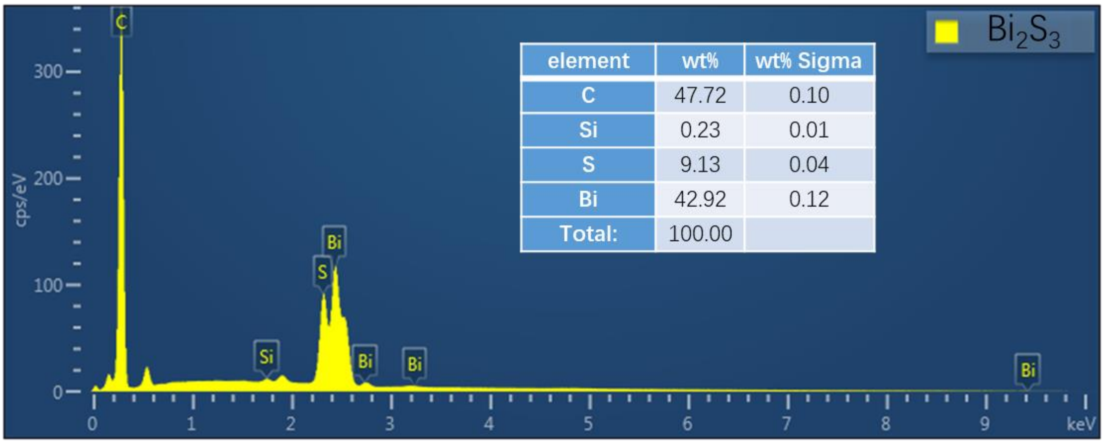

Figure S4. EDS spectra of the as-prepared c-BS.

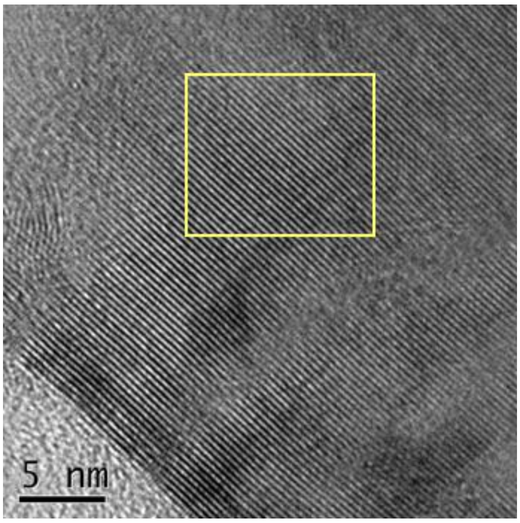

Figure S5 HRTEM image of c-BS.

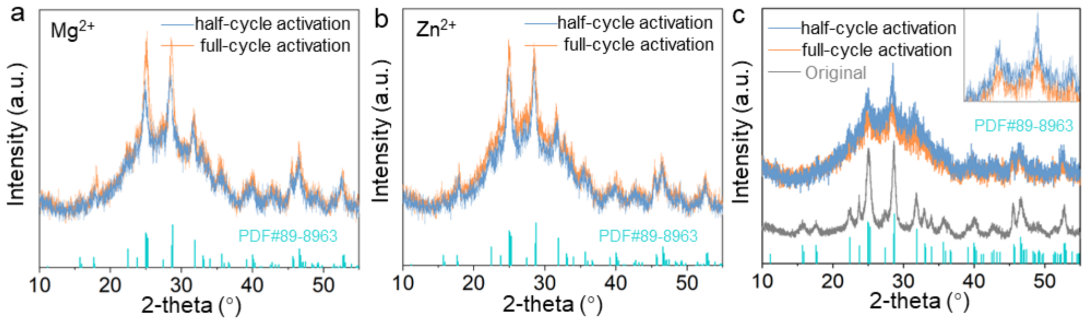

Figure S6. a) The XRD of Bi_2S_3 electrode under electrochemical activation at 200 mA g^{-1} using Mg^{2+} carriers. b) The XRD of Bi_2S_3 electrode under electrochemical activation at 200 mA g^{-1} using Zn^{2+} carriers. c) The XRD of Bi_2S_3 under electrochemical activation at 10 A g^{-1} using Cu^{2+} carriers.

Following a complete electrochemical activation, neither Mg^{2+} nor Zn^{2+} facilitates the amorphous transformation of c-BS (Figure S6a, b). The Cu^{2+} electrochemical activation process under high current density (10 A g^{-1}), where the amount of Cu^{2+} interaction with BS is decreased in a non-thermodynamically stable manner, demonstrates the obviously amorphous transition tendency of BS even a small amount of Cu^{2+} reaction with BS (Figure S6c).

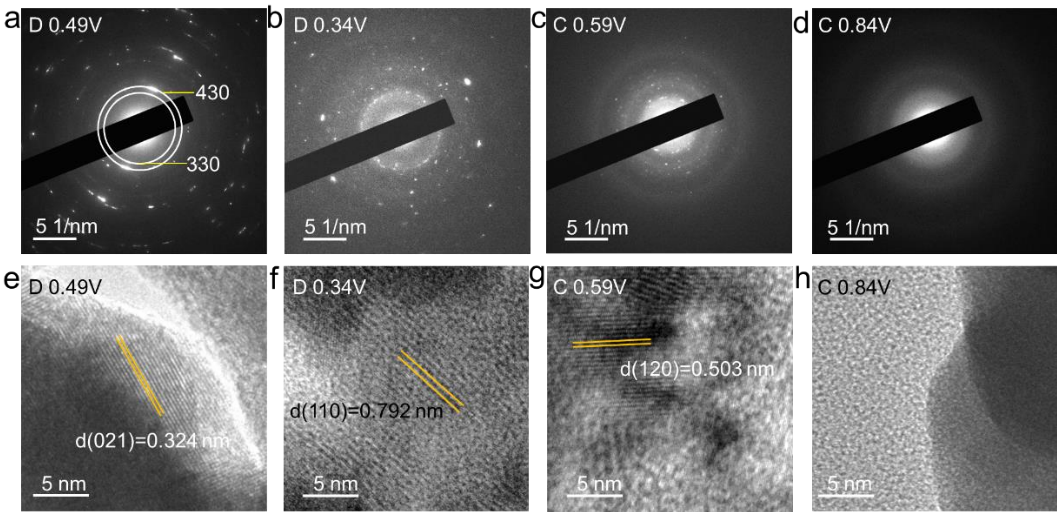

Figure S7. The SAED of Bi_2S_3 electrode at various electrochemical activation states: a) D 0.49 V (*vs.* SHE, discharge to 0.49 V), b) D 0.34 V (discharge to 0.34 V), c) C 0.59V (charge to 0.59 V), d) C 0.84 V (charge to 0.84 V). The HRTEM of Bi_2S_3 electrode at various electrochemical activation states: e) D 0.49 V, f) D 0.34 V, g) C 0.59 V, h) C 0.84 V.

The lattice spacing of 0.324 nm is distinctly observable at D 0.49 V, which corresponds to the crystalline plane (021) of c-BS, indicating its high degree of crystallinity. In the complete discharge state (D 0.34 V), the lattice stripe has become blurred, indicating that the crystallinity is deteriorating, and the subsequent copper ion extraction processes further evolve towards amorphization.

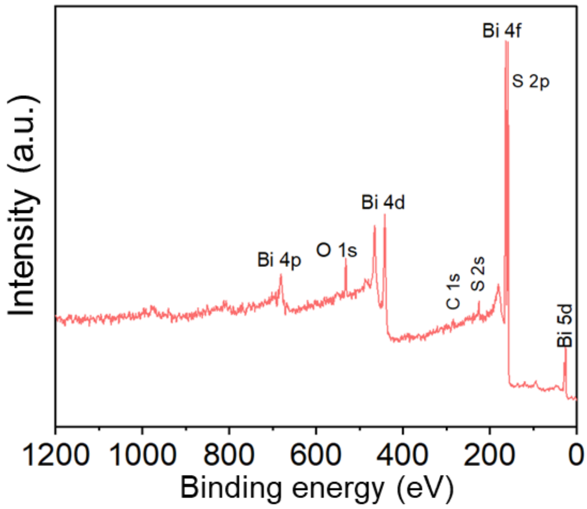

Figure S8. XPS survey spectrum of c-BS.

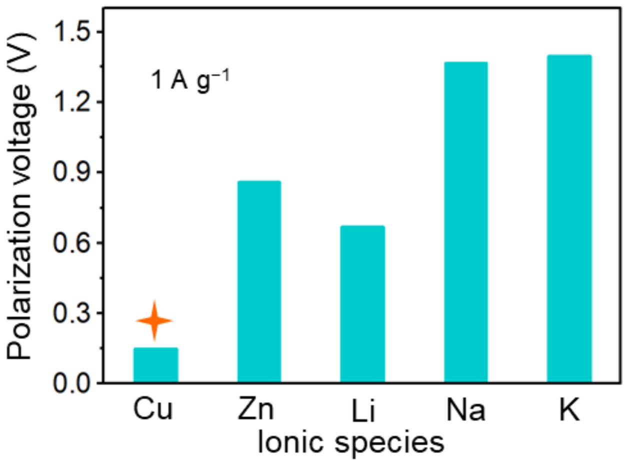

Figure S9. Contrast in the polarization voltage in different metal-ion batteries at the same current density.

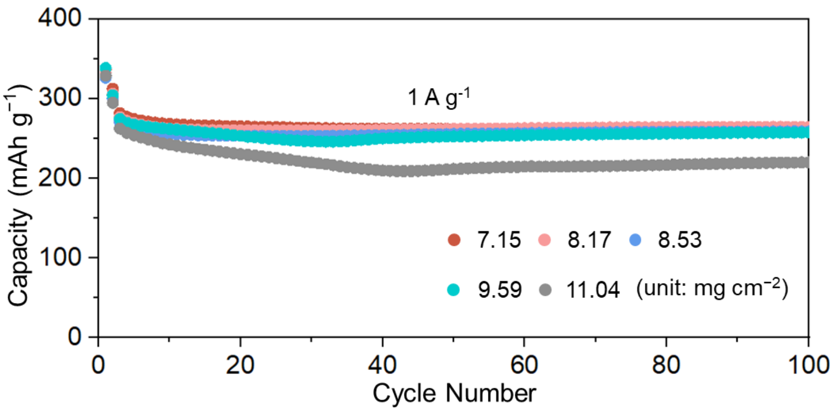

Figure S10 The capacity of a-BS electrode with different mass loadings at 1 A g^{-1} (200 µL electrolyte per cell).

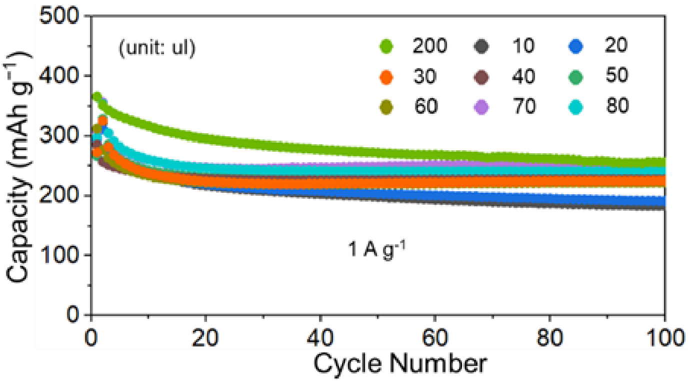

Figure S11. The discharge capacity of a-BS at varied electrolyte quantity from 10 to 200 μL (mass loading 1.8 mg cm^{-2}).

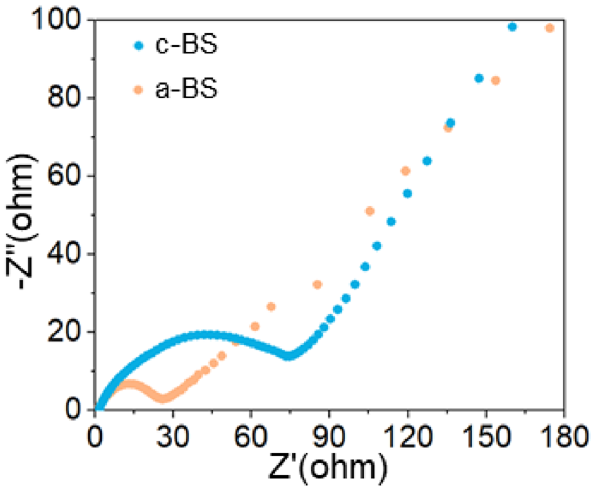

Figure S12. EIS of c-BS‖Cu and a-BS‖Cu battery.

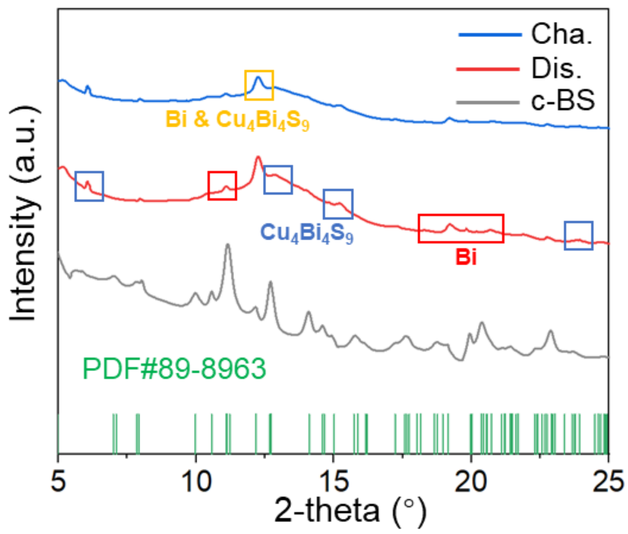

Figure S13. XRD pattern of electrode in different charge-discharge states.

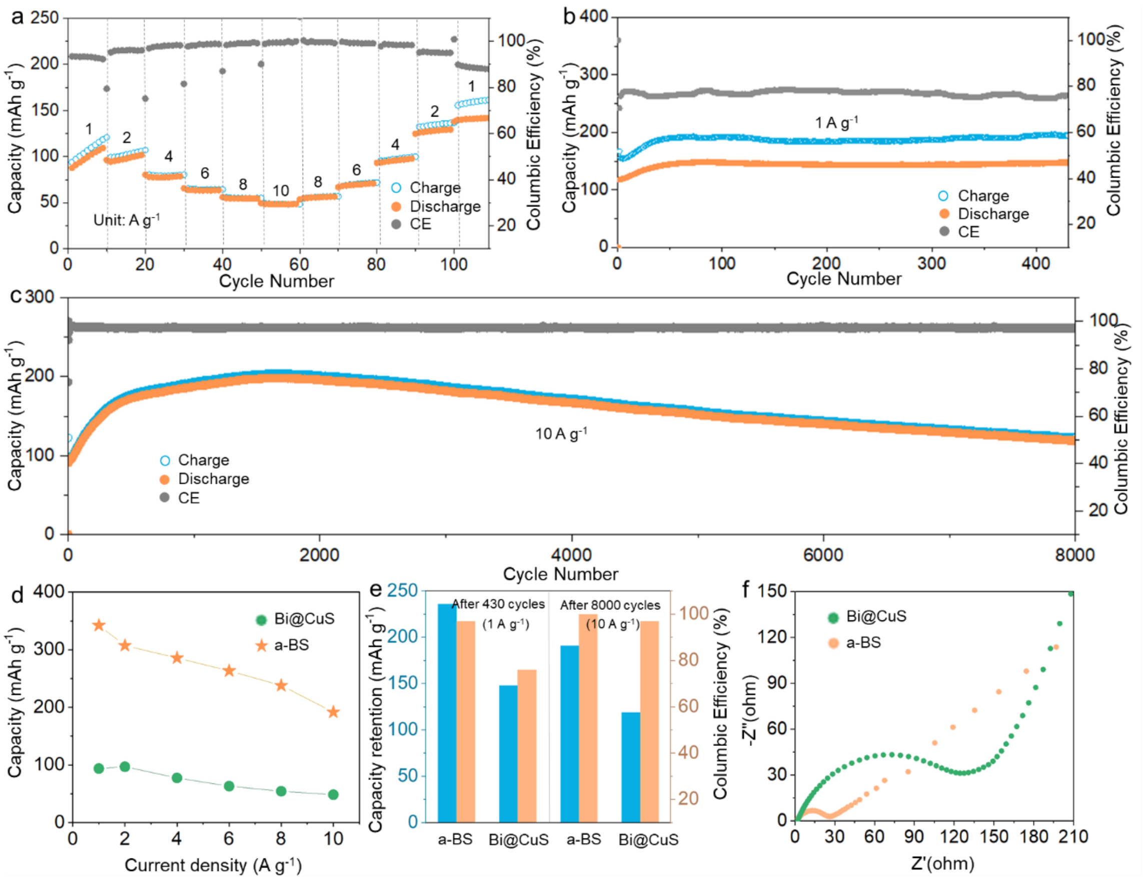

Figure S14. a) Rate performance of the Bi@CuS cathode. b) Cycling performance of the Bi@CuS cathode at 1 A g^{-1}. c) Cycling performance of the Bi@CuS cathode at 10 A g^{-1}. e) Capacity and corresponding Coulombic Efficiency of Bi@CuS‖Cu and a-BS‖Cu battery at various current densities. f) EIS of Bi@CuS‖Cu and a-BS‖Cu battery.

As a control, we proposed a Bi-loaded CuS composite cathode (Bi@CuS, with a molar ratio of 2:3, the same atomic ration as Bi_2S_3) to explore a deep conversion process based on the presumptive discharge/charge products. If the BS involved in a deep conversion, the discharge products will be Bi and copper sulfide. The deep conversion reaction of CuS with Cu^{2+} has been demonstrated in the literature[1]. Despite the conductive Bi promotes the conversion reaction, Bi@CuS cathodes manifest a poor rate performance, low Coulomb efficiency at 1 A g^{-1}, and a longer activation process (1600 cycles) at 10 A g^{-1}. When compared with the a-BS, the capacity (Bi@CuS cathodes) rapidly degrades after activation owing to the deactivation of the cathode material induced by the deep conversion reaction. We

further analyze the EIS data, and the Bi@CuS appears in a higher charge transfer resistance than the a-BS cathode. These results all demonstrate the advantages of amorphous structures and self-controlled moderate conversion in a-BS.

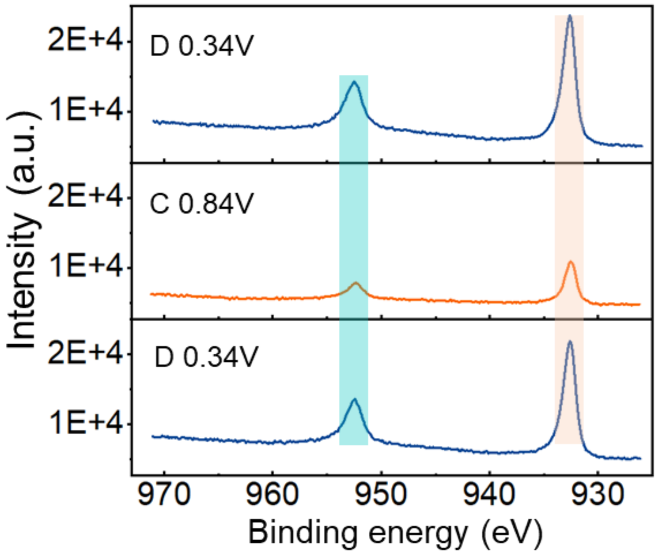

Figure S15. Ex situ XPS spectrum of Cu 2p of a-BS cathodes at the various discharging-charging states.

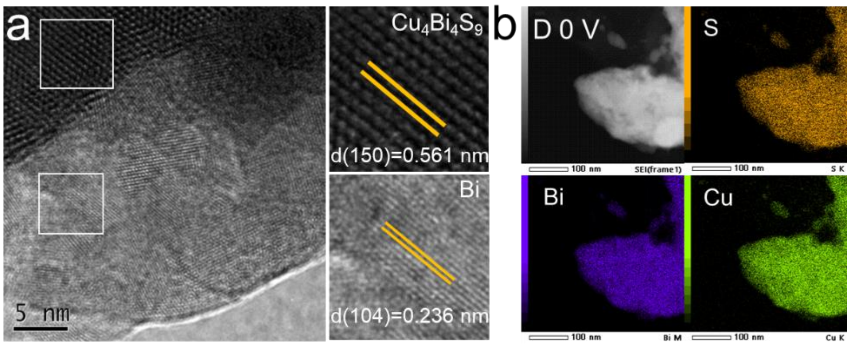

Figure S16. a) HRTEM image of a-BS cathodes at the fully discharged state after 50 cycles (200 mA g^{-1}). b) EDS of a-BS cathodes at the fully discharged state after 50 cycles (200 mA g^{-1}).

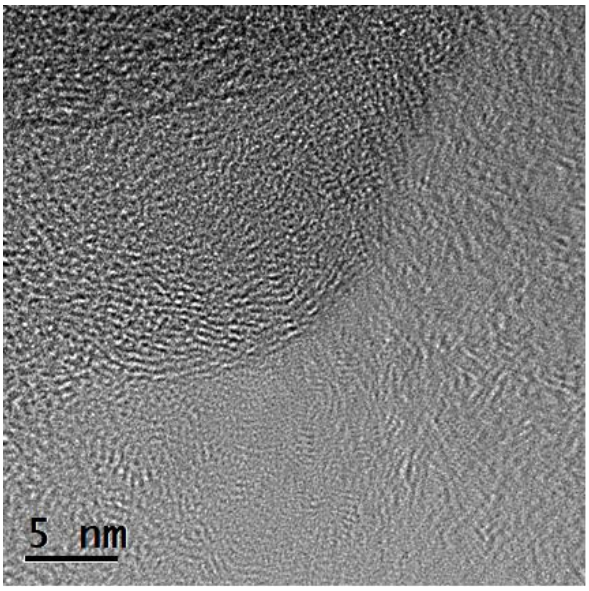

Figure S17. HRTEM image of a-BS cathodes at full charging state.

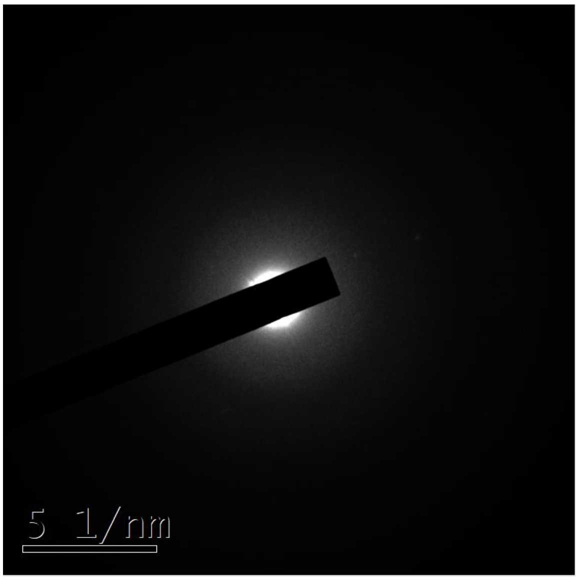

Figure S18.SAED image of a-BS cathodes at full charging state.

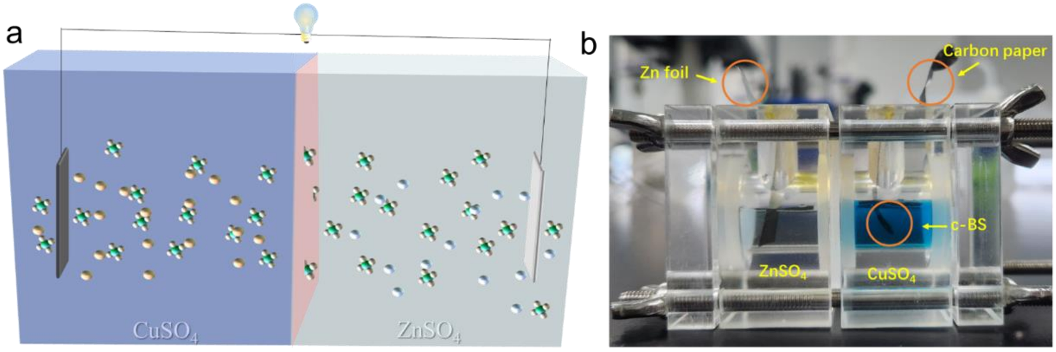

Figure S19. a) Schematic of the a-BS Cu^{2+}‖Zn^{2+} hybrid ions battery. b) Photograph of the a-BS Cu^{2+}‖Zn^{2+} hybrid ions battery.

Name	Cell type	Current density (A g^{-1})	Cycle number	Remaining Capacity (mAh g^{-1})	Ref. No.
a-BS	Cu//a-BS	10	8000	190.5	This work
Bi_2S_3 Nanoparticles	Zn//Bi_2S_3 Np	1	2000	75.6	[2]
Bi_2S_3/rGO	Na//Bi_2S_3/rGO	0.03	300	149.0	[3]
Bi_2S_3/C	Li// Bi_2S_3/C	0.1	100	765.0	[4]
$Bi_2S_{3-x}Se_x$/CF	Na//$Bi_2S_{3-x}Se_x$/CF	0.1	100	205.8	[5]
Bi_2S_3Nanoroll	Li//Bi_2S_3 Nr	1	600	541.0	[6]
PSS/Bi_2S_3	Zn//PSS/Bi_2S_3	2	5300	131.0	[7]
yolk@shell Bi_2S_3@C	Li//Bi_2S_3 SC	1	700	501.0	[8]
Bi_2S_3@rGO	K//Bi_2S_3@rGO	0.2	150	100.8	[9]
Bi_2S_3/TiO_2@PPy	Na//Bi_2S_3/TiO_2@PPy	10	2500	149.5	[10]
Bi_2O_3-Bi_2S_3	Mg//BO-BS	1.33	5000	55.1	[11]
Bi_2S_3/C yolk-shell	Na// Bi_2S_3/C	0.125	300	282	[12]

Bi_2S_3/Bi_2Se_3 vdWHs	K//Bi_2S_3/Bi_2Se_3 vdWHs	0.5	1000	201.7	[13]

Table S1. Comparsion of this work with reported Bi_2S_3-based secondary ion batteries

Reference

[1] Y. Wang, D. Chao, Z. Wang, J. Ni, L. Li, *ACS Nano* **2021**, *15*, 5420-5427.

[2] T. Xiong, Y. Wang, B. Yin, W. Shi, W. S. V. Lee, J. Xue, *Nanomicro Lett* **2019**, *12*, 8.

[3] J. H. Yu, C.-H. Jo, H. J. Kim, S.-T. Myung, *Energy Storage Materials* **2021**, *38*, 241-248.

[4] W. Chai, F. Yang, W. Yin, S. You, K. Wang, W. Ye, Y. Rui, B. Tang, *Dalton Trans* **2019**, *48*, 1906-1914.

[5] X. Chen, Y. Hong, X. Ge, C. Li, X. Miao, P. Wang, Z. Zhang, L. Yin, *Journal of Alloys and Compounds* **2020**, *825*.

[6] Z. Zou, Q. Wang, K. Zhu, K. Ye, G. Wang, D. Cao, J. Yan, *Small* **2022**, *18*, e2106673.

[7] Y. Zhao, L. Ma, Y. Zhu, P. Qin, H. Li, F. Mo, D. Wang, G. Liang, Q. Yang, W. Liu, C. Zhi, *ACS Nano* **2019**, *13*, 7270-7280.

[8] L. Zhao, H. H. Wu, C. Yang, Q. Zhang, G. Zhong, Z. Zheng, H. Chen, J. Wang, K. He, B. Wang, T. Zhu, X. C. Zeng, M. Liu, M. S. Wang, *ACS Nano* **2018**, *12*, 12597-12611.

[9] L. Yuan, Q. Zhou, T. Li, Y. Wang, Z. Liu, S. Chong, *Applied Energy* **2022**, *322*.

[10] H. Guan, X. Du, Y. Yi, X. Kang, K. Li, X. Pei, Z. Zhao, J. Zhang, D. Li, *ACS Appl Mater Interfaces* **2021**, *13*, 55051-55059.

[11] Q. Tang, Y. Song, X. Cao, C. Yang, D. Wang, T. Qin, W. Zhang, *Journal of Magnesium and Alloys* **2023**.

[12] H. Kim, D. Kim, Y. Lee, D. Byun, H.-S. Kim, W. Choi, *Chemical Engineering Journal* **2020**, *383*.

[13] Y.-Y. Hsieh, H.-Y. Tuan, *Energy Storage Materials* **2022**, *51*, 789-805.
